# Supplementary material for: Endothelial-Specific Molecule 1 Inhibition Lessens Productive Angiogenesis and Tumor Metastasis to Overcome Bevacizumab Resistance
Source: Cancers (Basel). 2022 Nov 18;14(22):5681. doi: 10.3390/cancers14225681 (PMC9688485; doi:10.3390/cancers14225681)

1. Figure S1: Western blot of MMP9 in MDA−MB−231−S, MDA−MB−231−R, MDA-−MB−231−S ^{ovESM1} and MDA−MB−231−R ^{shESM1} cells in Figure 3j

Loading order (left to right):

MDA-MB-231-S, MDA-MB-231-S ^{ovESM1}, MDA-MB-231-R, and MDA-MB-231-R ^{shESM1}

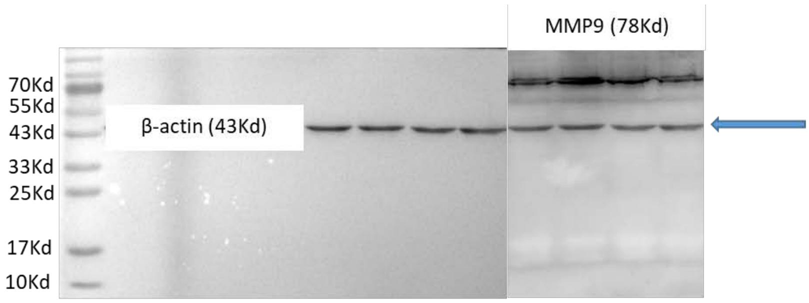

After the bloting of β-actin were exposured, we reblocked the PVDF membrane and continued to expose MMP9 in this PVDF membrane, so there is an bloting of MMP9 here.

2. Figure S2: RelA or RelB was overexpressed in MDA−MB−231−S cells and detected by western blotting in Figure 4c:

Loading order (left to right): MDA-MB-231-S, MDA-MB-231-S ^{ovRelA}

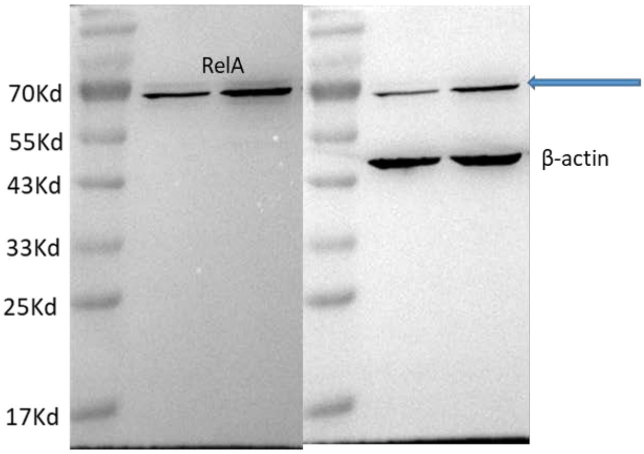

After the bloting of RelA were exposured, we reblocked the PVDF membrane and continued to expose β-actin in this PVDF membrane, so there is an bloting of Rel A here.

Loading order (left to right):

MDA-MB-231-S, MDA-MB-231-S ^{ovRelB}

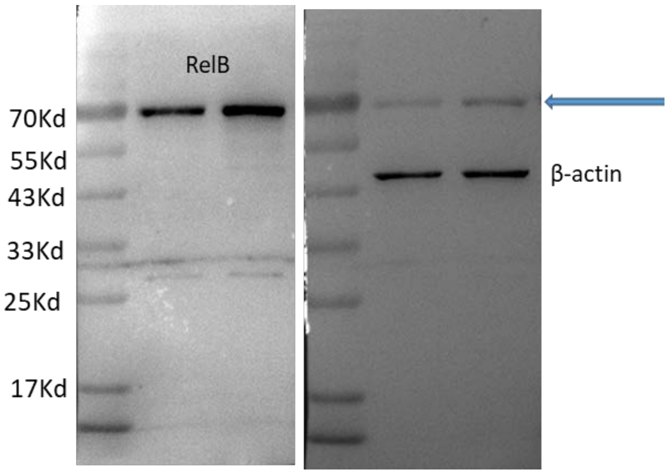

After the blotings of RelB were exposed, we reblocked the PVDF membrane and continued to expose β-actin in this PVDF membrane, so there is a bloting of RelB here.

3. Figure S3: Western blotting for DLL4 in HUVEC cells with or without human ESM1 added in Figure 6c:

Loading order (left to right):

HUVEC, HUVEC-ESM1

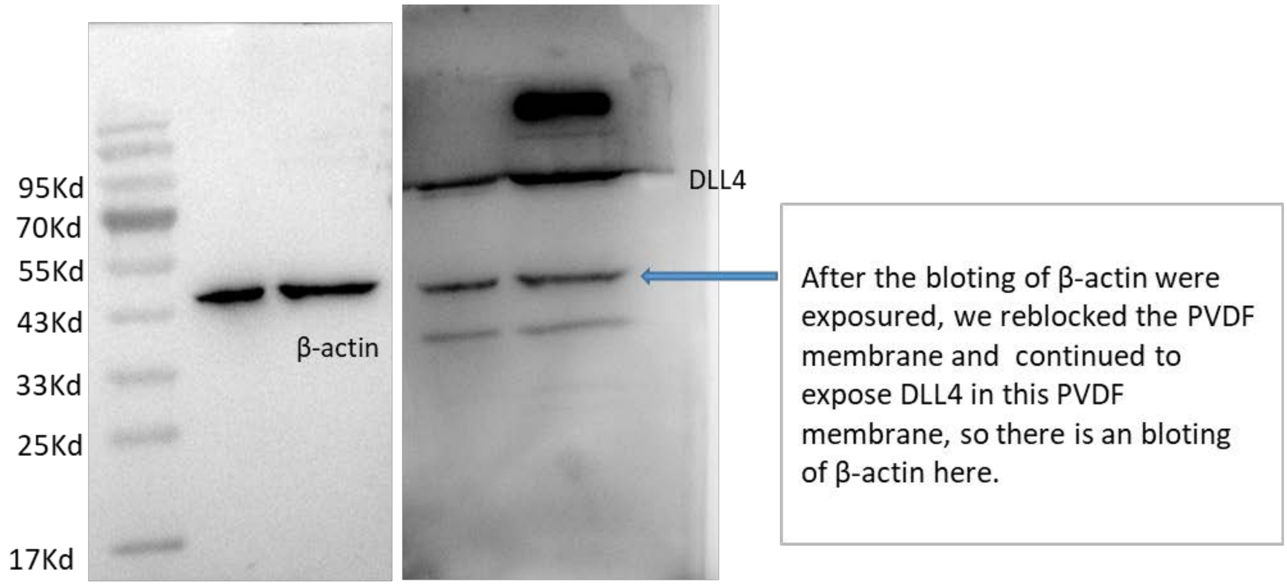

4. Figure S4: Western blotting for DLL4 in MDA−MB−231−S, MDA−MB−231−S ^{ovESM1}, MDA−MB−231−R in Figure 6d:

Loading order (left to right):

MDA-MB-231-S, MDA-MB-231-S ^{ovESM1}, MDA-MB-231-R

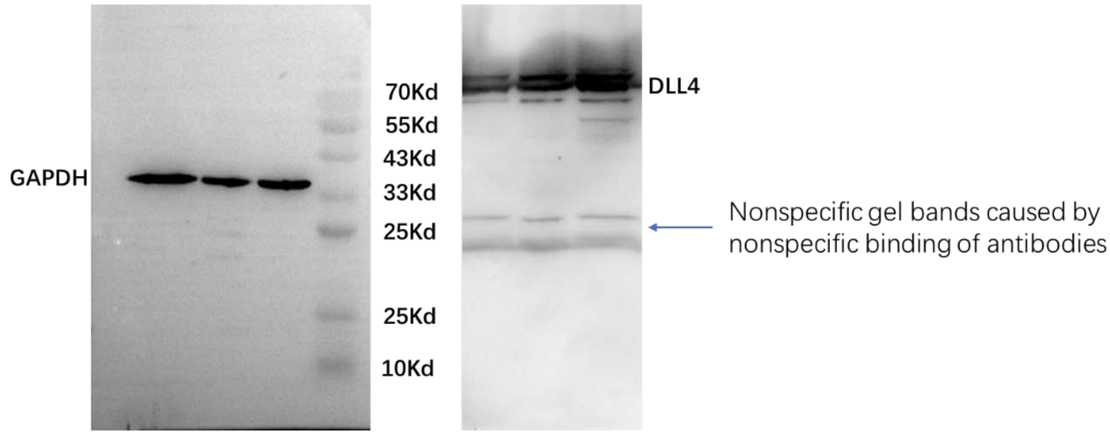

5. Figure S5: Western blotting for DLL4 in MDA−MB−231−R and MDA−MB−231 −R$^{shESM1\#1-3}$ in Figure 6e:

Loading order (left to right):

MDA-MB-231-R, MDA-MB-231-R$^{shESM1\#1}$, MDA-MB-231-R$^{shESM1\#2}$, MDA-MB-231-R$^{shESM1\#3}$

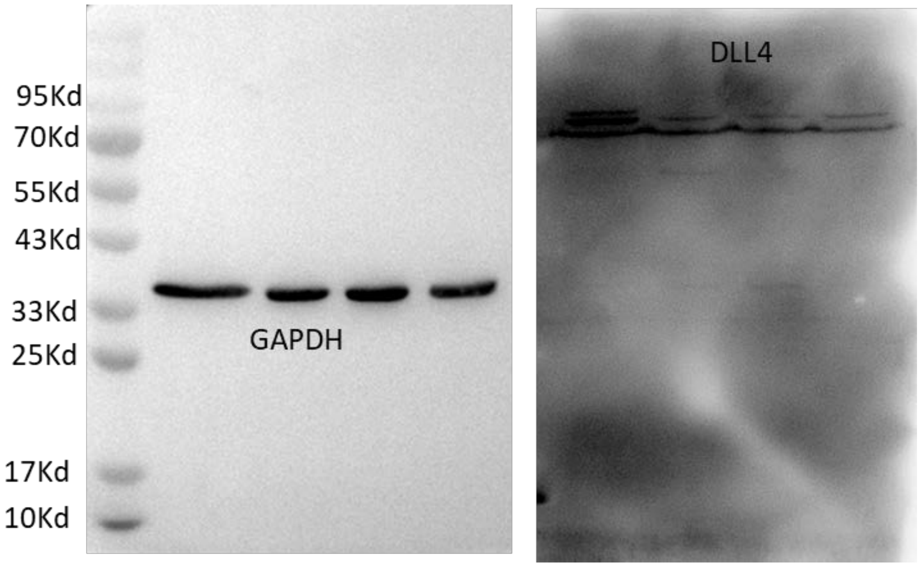

6. Figure S6: Western blotting for analyzing the expression of ESM1 deleted or not by the Crispr−Cas9 system in MDA−MB−231−R cells in Figure 7a:

Loading order (left to right):

MDA-MB-231-R, MDA-MB-231-R^{ESM1ko}

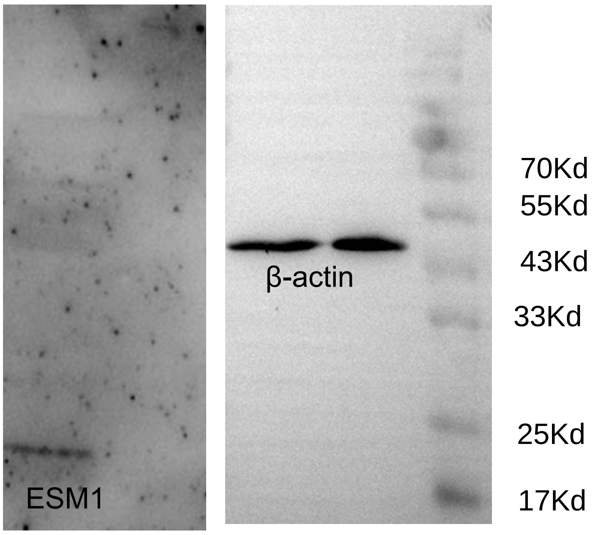

Supplement: Supplementary file 1 [file cancers-14-05681-s001.zip › cancers-2001562-supplementary.pdf]
